# *Rosa laevigata* Attenuates Allergic Asthma Exacerbated by Water-Soluble PM by Downregulating the MAPK Pathway

**DOI:** 10.3389/fphar.2022.925502

**Published:** 2022-06-28

**Authors:** Hyun Min Ko, Seung-Han Choi, Wona Jee, Seung-Hyeon Lee, Doil Park, Ji Hoon Jung, Beom-Joon Lee, Kwan-Il Kim, Hee-Jae Jung, Hyeung-Jin Jang

**Affiliations:** ^1^ College of Korean Medicine, Kyung Hee University, Seoul, South Korea; ^2^ Department of Science in Korean Medicine, Graduate School, Kyung Hee University, Seoul, South Korea; ^3^ Department of Biological Science in Korean Medicine, Graduate School, Kyung Hee University, Seoul, South Korea; ^4^ Division of Allergy, Immune and Respiratory System, Department of Internal Medicine, College of Korean Medicine, Kyung Hee University, Seoul, South Korea; ^5^ Department of Clinical Korean Medicine, Graduate School, Kyung Hee University, Seoul, South Korea

**Keywords:** *Rosa laevigata*, particulate matter, asthma, inflammation, IgE, cytokines, MAPK pathway

## Abstract

Exposure to water-soluble particulate matter (WPM) containing heavy metals can cause severe inflammatory responses and trigger and exacerbate the onset of asthma. As a follow-up study of *Rosa laevigata* (RL), this study analyzed the therapeutic effects and mechanisms of oral and intratracheal administration of RL and demonstrated anti-inflammatory effects in asthma models. Worse T-helper cell type 2 (Th2)-related inflammatory and pro-inflammatory responses were observed after simultaneous challenge with ovalbumin (OVA) and WPM. To establish a model of asthma exacerbated by WPM, BALB/c mice were sensitized with OVA + aluminum hydroxide and challenged with OVA + WPM. To confirm the therapeutic efficacy of RL, it was administered both orally and intratracheally. Histopathological analysis of H&E staining confirmed that oral and intratracheal administration of RL alleviated inflammatory cell infiltration in the airways aggravated by OVA + WPM. RL effectively reduced the number of inflammatory cells obtained from the bronchoalveolar lavage fluid. In addition, enzyme-linked immunosorbent assay (ELISA) and multiplex analysis of serum samples confirmed that the administration of RL reduced the levels of immuno-globulin E (IgE), Th2-related cytokines, and pro-inflammatory cytokines. Furthermore, real-time PCR analysis of lung tissue samples confirmed that the release of MUC5AC (Mucin 5AC, Oligomeric Mucus/Gel-Forming) and pro-inflammatory cytokines was reduced by RL, and western blotting confirmed that the administration of RL reduced the phosphorylation of ERK and p38 in the MAPK pathway. In conclusion, oral and intratracheal administration of RL appears to have an anti-asthmatic effect by reducing the secretion of Th2-related cytokines, pro-inflammatory cytokines, and IgE by downregulating the MAPK pathway. Thus, RL has further demonstrated potential for development as an oral and inhaled therapeutic for asthma symptoms exacerbated by WPM exposure.

## Introduction

Asthma is a heterogeneous chronic respiratory disease characterized by pathological features, such as chronic airway inflammation, airway hypersensitivity, reversible airway obstruction, airway remodeling, and vasodilation and is one of the most serious diseases worldwide (Bousquet et al., 2000). The prevalence of asthma is increasing in many parts of the world; according to the World Health Organization (WHO), it was reported that more than 300 million people were affected in 2017, and this is expected to increase to more than 400 million people in the future (Pawankar et al., 2012; Rehman et al., 2018; Stern et al., 2020).

Several translational studies have demonstrated that immunological, genetic, and environmental factors contribute to the risk of asthma development (Schoettler and Strek, 2020). Particulate matter (PM) pollutants, which have been one of the most central health issues in Korea in recent years, are a representative environmental factor that causes and aggravates asthma (Kim et al., 2013). Several studies have reported that long-term exposure to PM induces airway inflammation and oxidative stress, which can contribute to the onset and prevalence of respiratory and cardiovascular diseases, such as allergic asthma, chronic obstructive pulmonary disease (COPD), and pneumonia (Wong et al., 1999; Ge et al., 2018).

PM is mainly generated by soot, fossil fuel combustion, and automobile exhaust gas generated at industrial sites, such as construction sites and factories (de la Campa et al., 2010; Soleimani et al., 2018). It varies with time and space, and is a complex substance in which inorganic compounds such as metals and ions, organic compounds such as polycyclic aromatic hydrocarbon (PAH), and biological substances such as pollen and bacteria are mixed in various ways (Valavanidis et al., 2008). Water-soluble PM (WPM, mainly transition metals) can induce oxidative stress by influencing the generation of intracellular free radicals, which can worsen lung damage (Velali et al., 2016a; Zhao et al., 2019b). In addition, WPM promotes the expression of pro-inflammatory cytokines and chemokines by activating the NF-kB and MAPK pathways, which are closely related to multiple inflammatory responses, and aggravates the onset of allergic asthma by enhancing the infiltration of inflammatory cells in the lungs (Zhao et al., 2020b). There is also abundant evidence supporting an association between WPM and asthma, including that WPM induces pulmonary fibrosis via TGF-β1/Smad3 signaling in a murine model of asthma (Wu et al., 2021b). Therefore, as interest in the worsening of respiratory health due to PM increases, many researchers around the world are trying to discover treatments for various respiratory diseases that are aggravated by PM, such as asthma and COPD. Among the various treatment material candidates, natural materials have excellent therapeutic functions, have relatively few side effects, and are actively being researched.

*Rosa laevigata* (RL), which is mainly distributed in southern China and Asia, belongs to the Rosaceae family. RL fruit is edible and has been used in traditional medicine for the treatment of various diseases, including kidney disease and diarrhea (Qu et al., 2015). According to recent research, numerous lignans with antioxidant activity and compounds such as flavonoids, saponins, and tannins have been isolated from the fruits of RL (Li et al., 2012; Zhang et al., 2013d; Dong et al., 2015). In addition, it has been reported that the rich flavonoid extract of RL fruit is effective in preventing and treating neurodegenerative diseases (Liu et al., 2014; Liu et al., 2018), cerebral ischemia/reperfusion injury (Zhao et al., 2016; Liu et al., 2018), hyperlipidemia (Liu et al., 2010), non-alcoholic fatty liver disease (NAFLD) (Zhang et al., 2013e), and acute liver injury (Zhang et al., 2013b). In addition, we first confirmed in A549 cells that RL alleviated the inflammatory response in the lungs induced by exposure to PM10 by reducing cytokines such as COX-2 and IL-1β, IL-6, IL-13, and IL-17 via the MAPK and NF-kB pathways (Ko et al., 2020). In addition, we demonstrated the effectiveness of RL in treating allergic asthma via relieving the Th2 cell response and IgE secretion following intratracheal administration of RL in a mouse allergic asthma model (Lee et al., 2020). In addition, quercetin, which is the most common and representative flavonoid in RL extract, and tormentic acid and epicatechin, which exhibit anti-inflammatory effects, have been confirmed through LS-MS analysis. However, the anti-inflammatory mechanism of RL has been verified only in the inflammatory cell model induced by EGF or PM10. In particular, the anti-asthmatic effect and treatment mechanism of RL when administered orally have not yet been clearly elucidated. Therefore, as part of a follow-up study, we investigated the effectiveness and treatment mechanisms of RL in alleviating the Th2 cell response and acute airway inflammatory response after oral and intratracheal administration into mice with asthma exacerbation caused by PM exposure.

## Materials and Methods

### Reagents and Kits

OVA (Product no. A5503), hematoxylin (Product no. H3136), and eosin (Product no. E4382) were purchased from Sigma-Aldrich (St. Louis, MO, United States). Rabbit anti-phospho-extracellular signal-regulated kinase (ERK)1/2 (Product no. #9101), anti-ERK1/2 (Product no. #9102), anti-phospho p38 (Procudt no. #9211), and anti-p38 antibodies (Product no. #9212) were purchased from Cell Signaling Technology (CST Danvers, MA, United States). Mouse anti-β-actin (Product no. SC-47778), goat anti-mouse IgG-horseradish peroxidase (HRP, Product no. SC-2005), and goat anti-rabbit IgG-HRP (Product no. SC-2004) antibodies were purchased from Santa Cruz Biotechnology (Santa Cruz, CA, United States).

### Preparation of *Rosa laevigata* Extract

RL extract was obtained from PHARVIS R&D KOREA (Seoul, Korea). For the extraction, dried RL and purified water were mixed, extracted and filtered. Finally, RL extract was collected in the form of a dried extract powder.

### Preparation of Water-Soluble Particulate Matter and Measurement of Components

PM was collected by vehicles that drove in downtown Seoul for 6 months from 16 December 2019 to 17 June 2020, using a cabin filter (BOSCH, Karlsruhe, GERMANY) capable of blocking PM2.5. After removing the large particles filtered through the cabin filter for particle extraction, the filter was cut, immersed in Millipore water, and ultrasonic extraction was performed for 1 h using a sonicator on ice. The particulate matter extract was centrifuged at 12,000 rpm and 4°C, and the supernatant was filtered to remove insoluble particles using a 0.45 µm polytetrafluoroethylene (PTFE) filter syringe. Thereafter, it was lyophilized and stored at −80°C until use. The content of metal elements (Al, As, Be, Cd, Co, Cr, Cu, Fe, Mn, Mo, Se, Sr, Ti, Ni, Pb, and Zn) in the WPM extract was measured using LC-inductively coupled plasma mass spectrometry (ICP-MS, Agilent Technologies, Japan). The results are listed (Supplementary Table S1).

### Establishment of a Water-Soluble Particulate Matter-Exacerbated Mouse Model of Asthma and *Rosa laevigata* Treatment

Six-week-old female BALB/c mice were purchased from Daehan Biolink (DBL, Chungcheongbuk-do, Republic of Korea). This study was conducted in strict accordance with the ethical guidelines of Kyung Hee University. The animal study protocols were approved by the Institutional Animal Care and Use Committee (IACUC) of Kyung Hee University [confirmation number KHUASO-(SE)-20-063]. The asthma exacerbation model was slightly modified based on the OVA sensitized and challenged mouse model and was accompanied or followed by intranasal instillation of WPM solution concurrently with OVA injection. Briefly, a week after acclimatization, mice were randomly divided into the following seven groups: 1) Control; 2) OVA; 3) OVA + WPM; 4) OVA + WPM + RL oral gavage low dosage (ROL); 5) OVA + WPM + RL oral gavage high dosage (ROH); 6) OVA + WPM + RL intratracheal administraion low dosage (RIL); and 7) OVA + WPM + RL intratracheal administraion high dosage (RIH). All mice, except for the control group, were sensitized on days 0 and 14 by intraperitoneal injections of 200 µL of 2 mg aluminum hydroxide [Al(OH)3] and PBS (1:1) containing 100 µg OVA (Sigma-Aldrich), while the control group received 2 mg Al(OH)3 and PBS. For days 21–26, mice were challenged with 100 µL of OVA (50 µg) and WPM (600 µg) *via* nasal instillation. Normal control mice were challenged with PBS and OVA-only mice were challenged with OVA. After the last OVA + WPM challenge, the mice in the OVA + WPM group were instilled intranasally 2 times with WPM solution (600 µg/50 µL) at 6 h intervals. The mice in the control and OVA groups were instilled intranasally 2 times with 50 µL PBS at 6 h intervals. On day 27, all the mice were anesthetized with isoflurane after intranasal instillation. In parallel, the ROL and ROH groups were administered orally once a day at 50 mg/kg and 100 mg/kg, respectively, from the first sensitization (day 0) to the endpoint over 26 days. PBS was orally administered to the other groups. In addition, the RIL and RIH groups were intratracheally administered on day 3 and day 17 with 50 mg/kg or 100 mg/kg, respectively, according to the method of the previous study, and PBS was intratracheally administered to the other groups.

### Bronchoalveolar Lavage Fluid Collection and Analysis of Cell Composition

On day 27, the mice were anesthetized with intranasal isoflurane and sacrificed. Tracheostomy was performed to expose the trachea, and BALF was obtained by slow infusion and extraction of 1 ml ice-cold PBS. This procedure was repeated three times, and the lavages were pooled (mean volume, 2.0 ml). The recovered BALF was centrifuged at 1300 rpm for 10 min at 4°C. The cell pellet was resuspended in 1 ml PBS. A hemocytometer was used to count the total number of immune cells. The cells were then adhered onto a microscope glass slide using cytocentrifugation (Sandon, Waltham, MA, United States) and stained using the Diff-Quik staining kit (Product No. 26096, Electron Microscopy Science, Hatfield, PA, United States). A minimum of 200 cells were counted under a microscope, and based on general leukocyte morphology, were classified as macrophages, eosinophils, neutrophils, or lymphocytes.

### Enzyme-Linked Immunosorbent Assay for Immunoglobulin

Blood was collected from the mice and was left at room temperature (RT) for 1 h, and then centrifuged at ×1000 *g* for 15 min at 4°C to separate the serum. The total serum IgE level was analyzed using an enzyme-linked immunosorbent assay (ELISA) kit (Product No. ab157718, Abcam, Cambridge, United Kingdom), according to the manufacturer’s protocol.

### Multiplex for Cytokines and Chemokines

Serum was used to analyze the cytokine and chemokine levels. The levels of IL-4, IL-5, TNF-α, and eotaxin were measured using a Milli-plex® mouse cytokine/chemokine MAP kit (Product No. MCYTOMAG-70K, EMD Millipore Corporation, Billerica, MA, United States) and a Bio-Plex MAGPIX Multiplex reader (Bio-Rad, CA, United States). All experiments were performed in accordance with the manufacturer’s instructions.

### Real-Time Quantitative Polymerase Chain Reaction

Quantification of the mRNA expression of MUC5AC, IL-1β, IL-6, and IL-17A was performed using real-time quantitative PCR (Polymerase chain reaction). Total RNA from the lung tissue was extracted using Hybrid-RTM (Product No. 301-101, GeneAll, South Korea) according to the manufacturer’s protocol. RNA yield was measured using a NanoDrop spectrophotometer (Thermo Fisher Scientific, Waltham, MA, United States). Single-strand cDNA was synthesized using the Maxime RT premix (Product No. 25082, iNtRON Biotechnology, South Korea) according to the manufacturer’s protocol, and real-time quantitative PCR was performed using an Applied Biosystems Step One System (Applied Biosystems, Foster City, CA, United States) using the Universal SYBR Green Master Mix (Product No. 4367659, Applied Biosystems, Foster City, CA, United States). In this study, quantification based on the relative expression of a target gene versus the GAPDH gene (2^−ΔΔCt^ method) was performed to determine the level of mRNA expression. The PCR primers that were used are listed below.

MUC5AC: (F) 5′-ACA​TTT​CCC​CAT​GCT​CCA​CAG​C-3′ and (R) 5′-GTG​GTG​GTA​TTA​GAC​TCC​TGG-3′.

IL-1β: (F) 5′-GTC​ACA​AGA​AAC​CAT​GGC​ACA​T-3′ and (R) 5′-GCC​CAT​CAG​AGG​CAA​GGA-3′.

IL-6: (F) 5′-GCC​TAT​TGA​AAA​TTT​CCT​CT-3′ and (R) 5′-GTT​TGC​CGA​GTA​GAT​CTC-3′.

IL-17A: (F) 5′-CCT​GGC​GGC​TAC​AGT​GAA​G-3′ and (R) 5′-TTT​GGA​CAC​GCT​GAG​CTT​TG-3′.

GAPDH: (F) 5′-CAT​GGC​CTT​CCG​TGT​TCC​TA-3′ and (R) 5′-GCG​GCA​CGT​CAG​ATC​CA-3′.

### Histological Examination of Lung Tissue

After sacrifice, the left lung was dissected and fixed with 10% formalin. Dehydrated tissues were embedded in paraffin blocks and cut into 4 μm slices. The embedded tissues were stained with hematoxylin and eosin (H&E). Histopathological features were monitored using an Olympus BX51 microscope (Olympus, Tokyo, Japan) equipped with a DP71 orbital camera (Olympus). Pulmonary histopathological changes were evaluated on a subjective score of 0–5 on randomized, blinded sections by three independent readers according to the following criteria: 0, normal; 1, very mild; 2, mild; 3, moderate; 4, marked; and 5, severe inflammation. The thickness of the lung bronchial epithelium was quantified using Image J software (NIH, Bethesda, MD, United States).

### Western Blotting

Total protein from the lung homogenate was extracted using radioimmunoprecipitation assay (RIPA) buffer with protease inhibitor cocktails. Protein concentrations were quantified using the Bradford assay. Proteins (20 µg) were separated in 10% SDS-PAGE and transferred onto nitrocellulose membranes. Membranes were blocked with 3% bovine serum albumin (BSA) at RT for 1 h and incubated with diluted primary antibodies against p-ERK, ERK, p-p38, p38, and β-actin at 4°C for overnight. After removing the primary antibodies, Tris-buffered saline with Tween 20 (TBST)-washed nitrocellulose membranes were incubated with horseradish peroxidase (HRP)-conjugated secondary antibodies from Santa Cruz Biotechnology at RT for 1 h. To quantify expression levels, membranes were reacted with enhanced chemiluminescence (ECL) reagents and detected using a chemiluminescence imaging system (YoungIn, Seoul, Korea).

### Statistical Analysis

Statistical analysis of the data was performed using GraphPad Prism 5 software package (GraphPad Software, San Diego, CA, United States). The comparison between multiple groups was analyzed by ANOVA Tukey test, and comparison between 1:1 groups was performed using the unpaired t test (one-tailed). *p* values lower than 0.05 were considered significant. All data are presented as the mean ± SEM.

## Results

### *Rosa laevigata* Reduces the Lungs and Spleens in Mice With Asthma Exacerbated by Water-Soluble Particulate Matter Exposure

**FIGURE 1:**
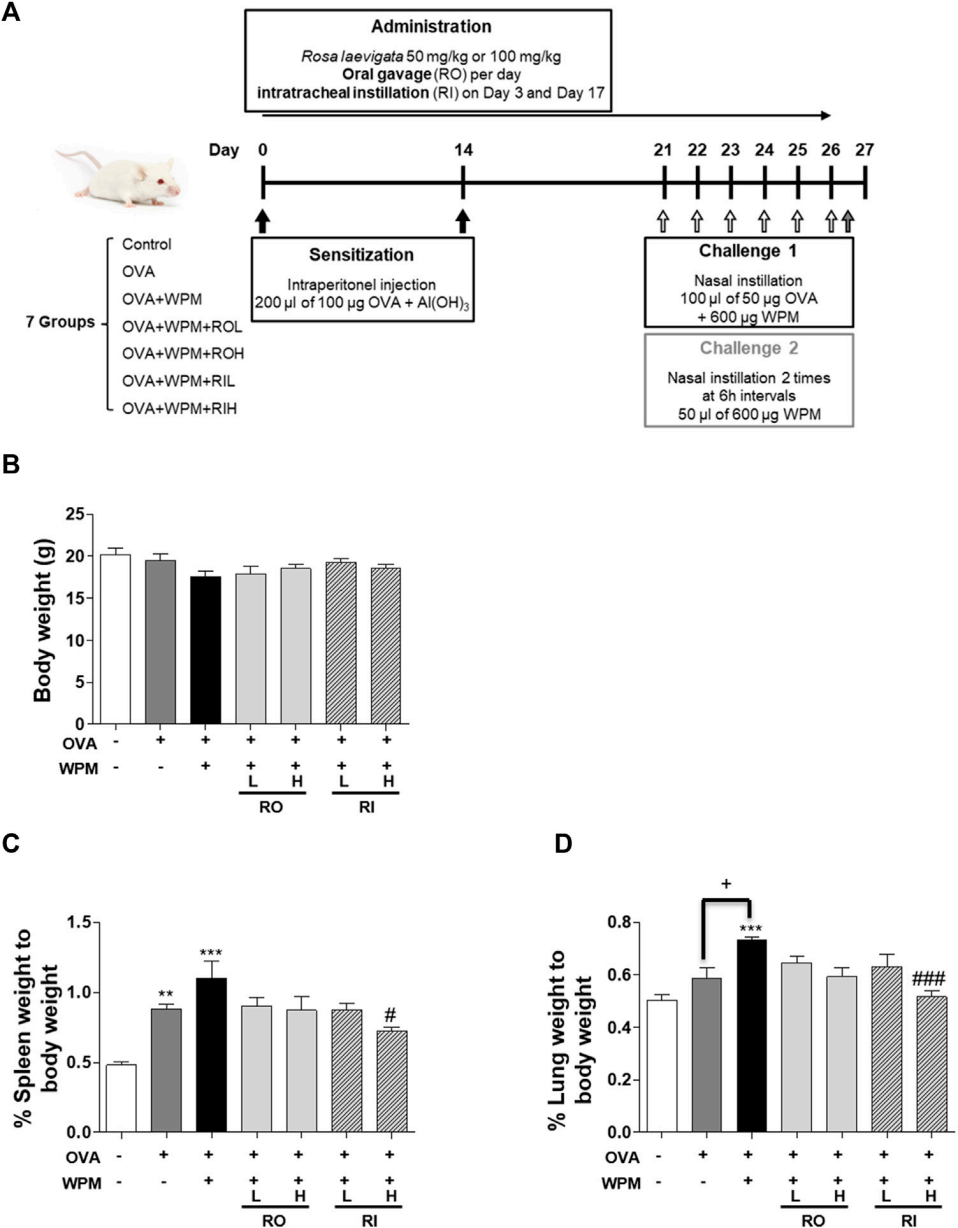
The effects of RL on organ weight in a mouse model of asthma exacerbated by WPM exposure. **(A)** A WPM-exacerbated mouse model of asthma and RL treatment was established. **(B**–**D)** The effects of RL on the weights of **(B)** the body, **(C)** spleen and **(D)** lungs of WPM-exacerbated mouse model. All data are presented as the mean ± SEM. *n* = 5/group. **p < 0.005 and ***p < 0.001 compared with the control group; ^#^ *p* < 0.05 and ^###^ *p* < 0.001 compared with the OVA + WPM group; ^+^ *p* < 0.05 compared with the OVA group.

A mouse model of WPM exacerbation in asthma is shown in Figure 1A. At the end of the experiment, there was no significant difference in the body weight between the groups (Figure 1B). However, the OVA and OVA + WPM treatment groups had higher relative weights of the spleen and lungs compared to those of the control group. In particular, the OVA + WPM treatment group showed significantly increased lung weights compared to those of the OVA treatment group (Figures 1C,D). However, the spleen and lung weights increased by OVA and WPM were decreased by oral and intratracheal administration of the RL extract.

### *Rosa laevigata* Alleviates Inflammatory Cell Infiltration in the Airways of Mice with Asthma Exacerbated by Water-Soluble Particulate Matter Exposure

**FIGURE 2:**
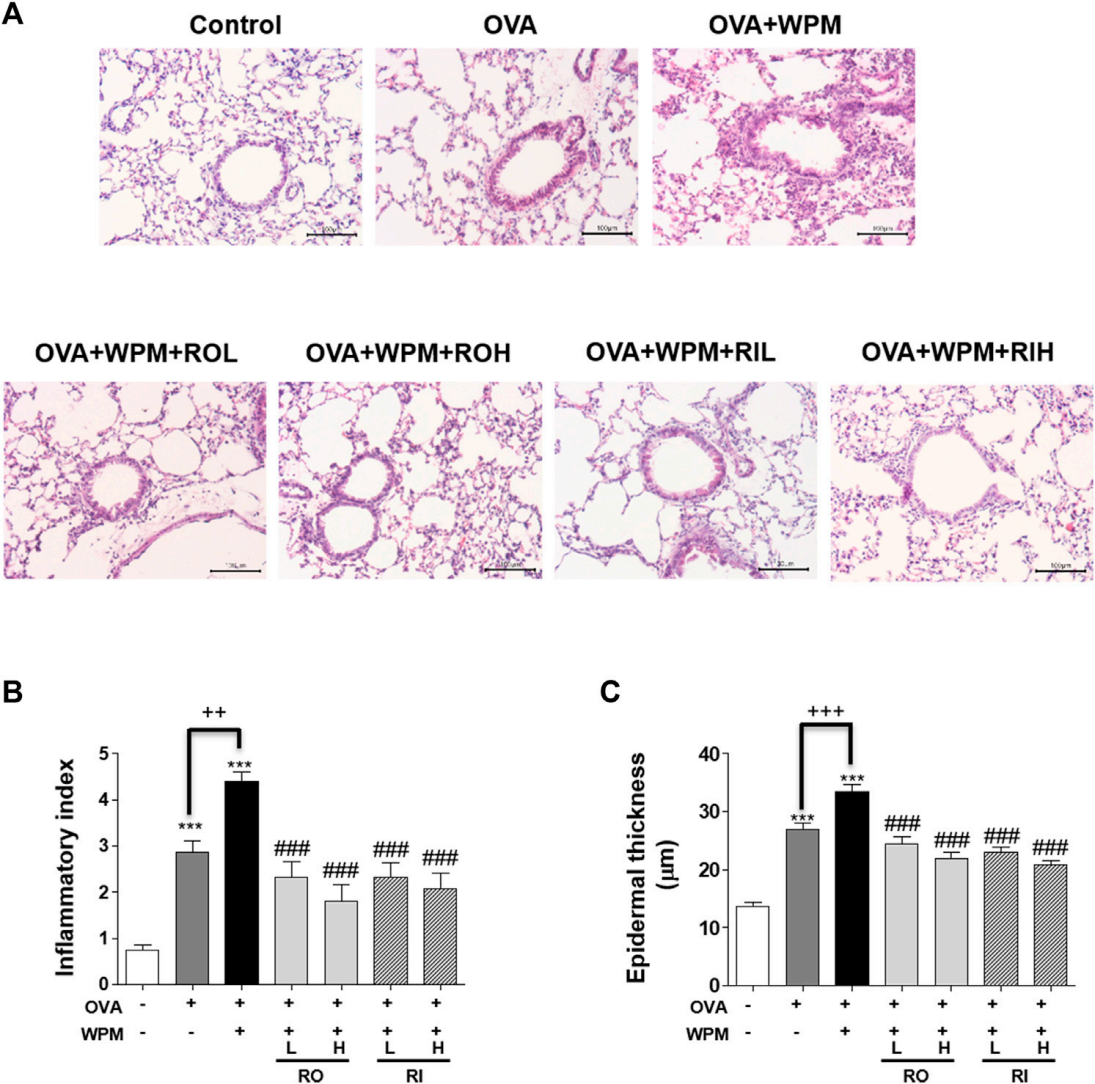
The effects of RL on histopathological changes in a WPM-exacerbated mouse model of asthma. Lung tissues were collected and fixed in 10% formalin. **(A)** Then the sections were cut and stained with H&E solution. **(B)** Inflammatory cell infiltration in lung tissues was scored as described in the Materials and Methods section. **(C)** The thickness of the lung bronchial epithelium was quantified using Image J software. All data are presented as the mean ± SEM. *n* = 4–5/group. ***p < 0.001 compared with the control group; ^###^ *p* < 0.001 compared with the OVA+WPM group; ^++^ *p* < 0.005 and ^+++^ *p* < 0.001 compared with the OVA group.

**FIGURE 3:**
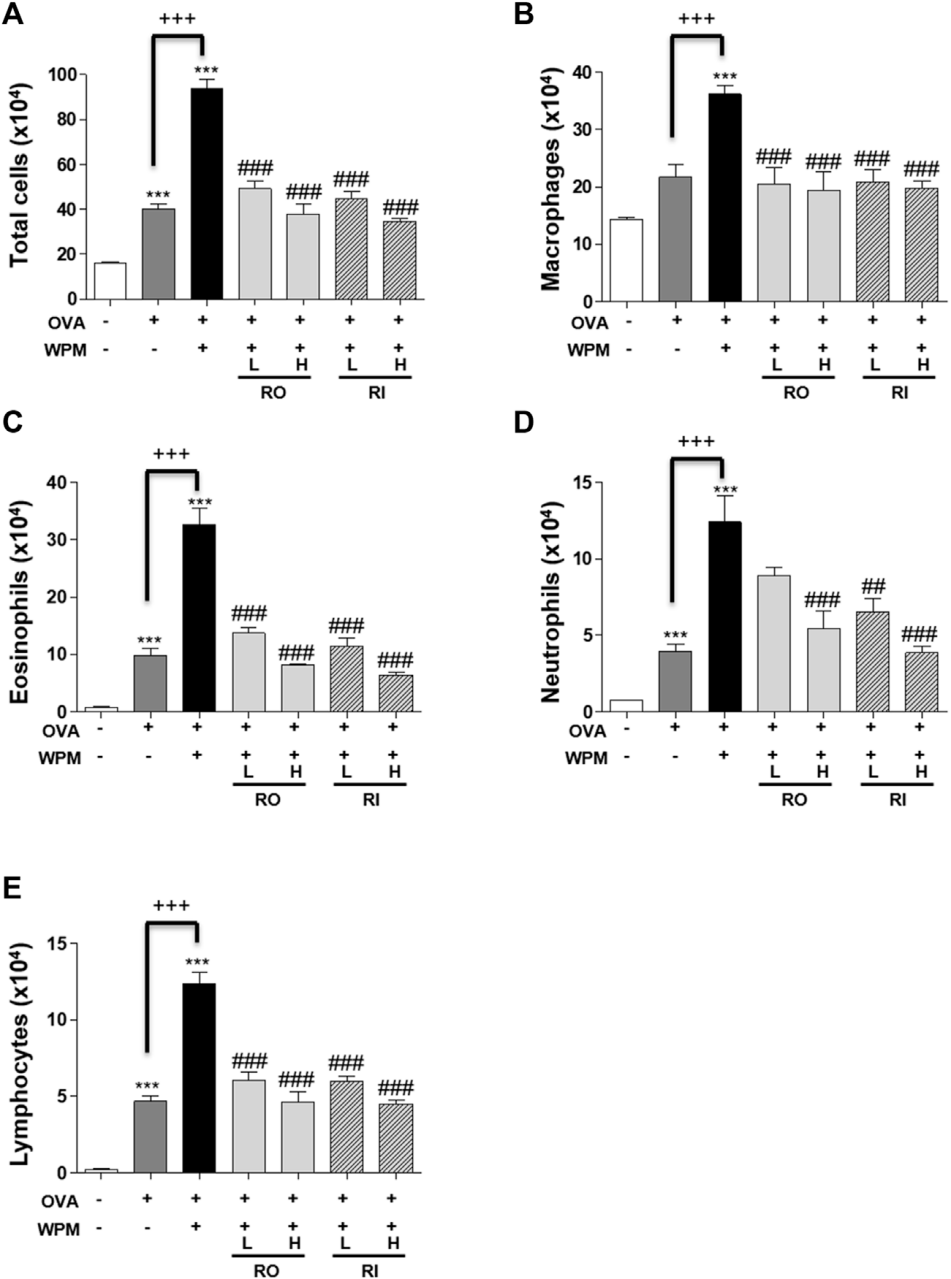
The effects of RL on inflammatory cell infiltration in a WPM-exacerbated mouse model of asthma. Cell population of **(A)** total cells, **(B)** macrophage, **(C)** eosinophils, **(D)** neutrophils and **(E)** lymphocytes in BALF. All data are presented as the mean ± SEM. *n* = 5/group. ***p < 0.001 compared with the control group; ^##^ *p* < 0.005 and ^###^ *p* < 0.001 compared with the OVA + WPM group; ^+++^ *p* < 0.001 compared with the OVA group.

We performed H&E staining of the lungs collected from mice in each group to analyze histological changes in the lungs (Figure 2A). As shown in Figures 2B,C, the degree of infiltration of inflammatory cells around the peribronchial and perivascular regions was more severe in the OVA + WPM group than in the control and OVA groups. Oral and intratracheal administration of RL significantly attenuated the infiltration of exacerbated inflammatory cells compared to that in the OVA + WPM group. In addition, since the increase in the number of inflammatory cells infiltrating the airways is a major biomarker for the development of allergic asthma, we investigated the total cell number and cell type in bronchoalveolar lavage fluid (BALF) 24 h after nasal administration of WPM (Figures 3A–E). Cells other than macrophages were hardly detected in the control group, whereas the number of macrophages, eosinophils, neutrophils, and lymphocytes was significantly increased in the OVA and OVA + WPM-treated groups compared to that in the control group. In addition, compared to the OVA group, the OVA + WPM treatment group showed a significant increase in the total number of cells and individual cells in the BALF. However, oral and intratracheal administration of RL effectively reduced the infiltration of macrophages, eosinophils, neutrophils, and lymphocytes exacerbated by OVA + WPM.

### *Rosa laevigata* Alleviates Serum Immunoglobulin Secretion Levels in Mice with Asthma Exacerbated by Water-Soluble Particulate Matter Exposure

**FIGURE 4:**
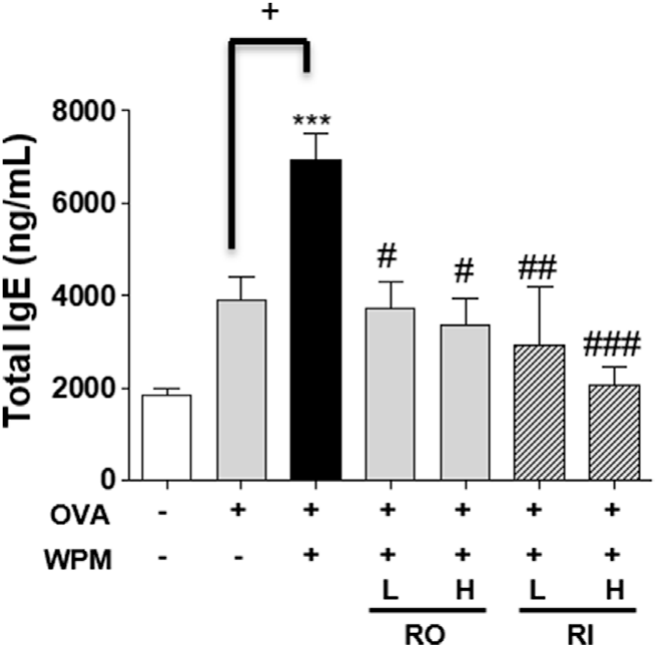
The effects of RL on allergic response in a WPM-exacerbated mouse model of asthma. The total IgE levels in serum were analyzed by ELISA. All data are presented as the mean ± SEM. *n* = 4/group. ***p < 0.001 compared with the control group; ^#^ *p* < 0.05, ^##^ *p* < 0.005 and ^###^ *p* < 0.001 compared with the OVA + WPM group; ^+^ *p* < 0.05 compared with the OVA group.

To investigate the effect of RL administration on OVA-induced systemic allergic reactions exacerbated by WPM, the total serum IgE levels were measured. As shown in Figure 4, the expression level of serum IgE was increased in the OVA and OVA + WPM groups compared to that in the control group. Specifically, it was confirmed that the level of serum IgE in the OVA + WPM group was significantly increased by approximately 1.78 times compared to that in the OVA group. However, oral and intratracheal administration of RL significantly and dose-dependently reduced the serum IgE levels exacerbated by OVA + WPM induction. In particular, when RL was administered intratracheally at a high dose, it was the most effective.

### *Rosa laevigata* Regulates the Secretion Level of Inflammatory Cytokines in the Serum of Mice with Asthma Exacerbated by Water-Soluble Particulate Matter Exposure

**FIGURE 5:**
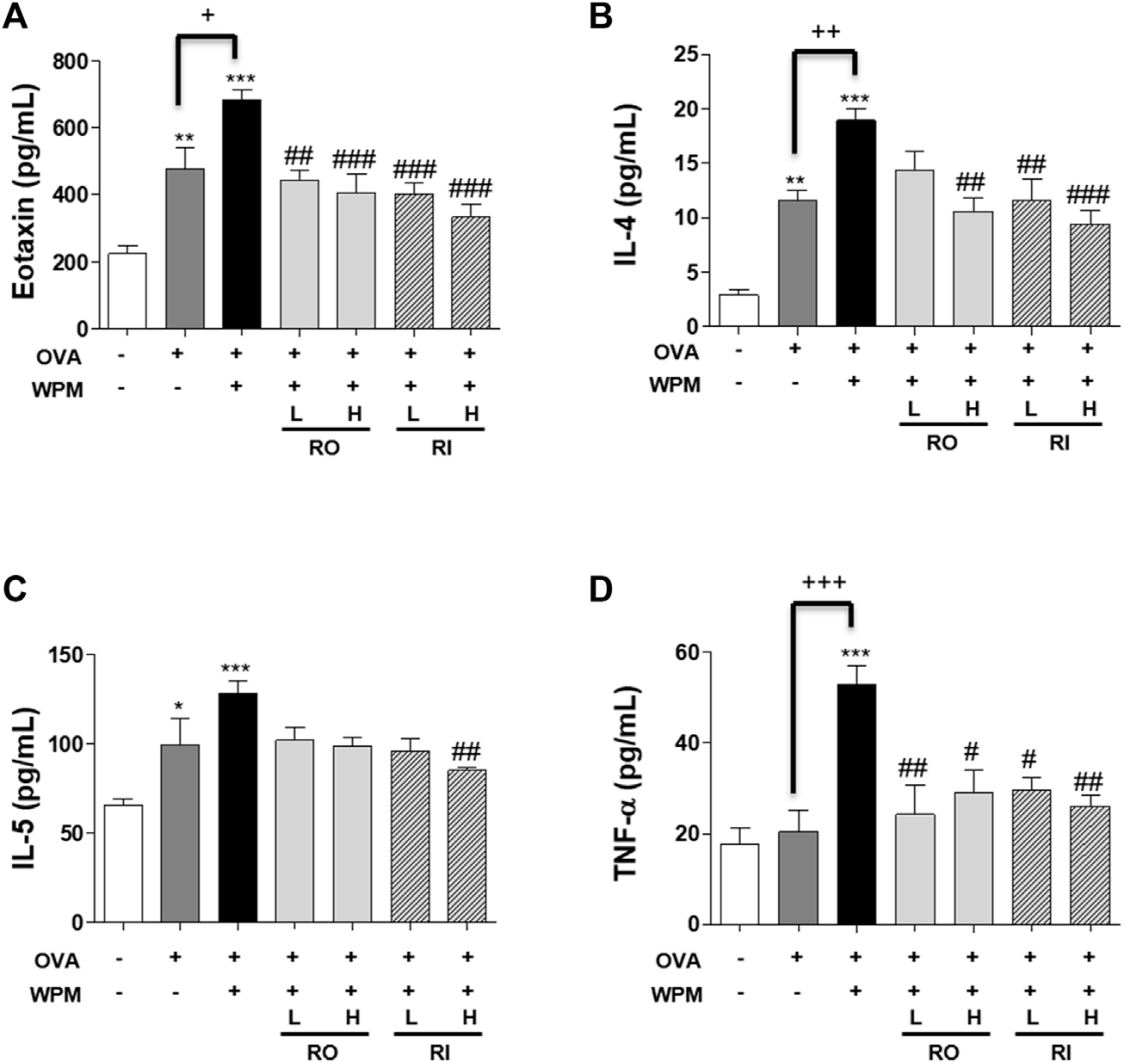
The effects of RL on the cytokine and chemokine levels in a WPM-exacerbated mouse model of asthma. The levels of **(A)** Eotaxin, **(B)** IL-4, **(C)** IL-5 and **(D)** TNF-α were analyzed using a Multiplex ELISA kit. All data are presented as the mean ± SEM. *n* = 5/group. *p < 0.05, **p < 0.005 and ***p < 0.001 compared with the control group; ^#^ *p* < 0.05, ^##^ *p* < 0.005 and ^###^ *p* < 0.001 compared with the OVA + WPM group; ^+^ *p* < 0.05, ^++^ *p* < 0.005 and ^+++^ *p* < 0.001 compared with the OVA group.

We also investigated the secretion levels of inflammatory cytokines and chemokines associated with allergic asthma in serum samples from mice with asthma exacerbated by WPM using Multiplex (Figures 5A–D). In the serum, Th2-related cytokines IL-4 and IL-5 and the chemokine eotaxin were significantly increased in the OVA and OVA + WPM groups compared to those in the control group. In addition, WPM exposure significantly increased the secretion of IL-4 and eotaxin compared to that in the OVA group. However, the secretion of Th2-related cytokines and chemokines was alleviated by oral and intratracheal administration of RL, and a more pronounced effect was observed when it was administered at high doses. In addition, the secretion level of TNF-α, a pro-inflammatory cytokine, was upregulated by about 3 times in the OVA + WPM group compared to the control, but oral and intratracheal administration of RL significantly inhibited TNF-α.

### *Rosa laevigata* Regulates mRNA Expression of MUC5AC and Pro-Inflammatory Cytokines in the Lungs of Mice with Asthma Exacerbated by Water-Soluble Particulate Matter Exposure

**FIGURE 6:**
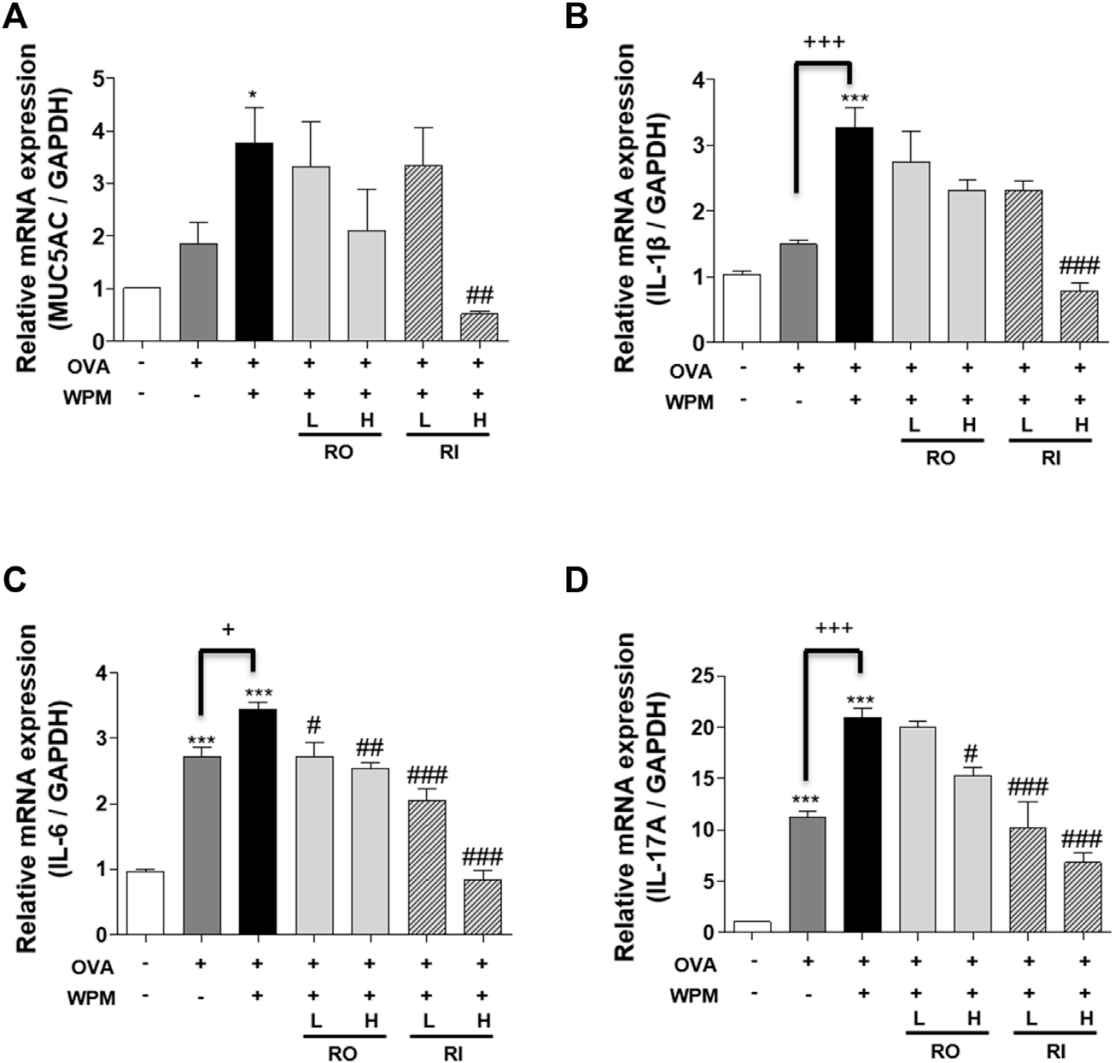
The effects of RL on the mRNA level of MUC5AC and pro-inflammatory cytokines in the lungs of WPM-exacerbated mouse model of asthma. **(A)** The mRNA levels of MUC5AC, and **(B**–**D)** inflammatory cytokines, **(B)** IL-1β, **(C)** IL-6 and **(D)** IL-17A were analyzed by real-time PCR. All data are presented as the mean ± SEM. *n* = 5/group. *p < 0.05 and ***p < 0.001 compared with the control group; ^#^ *p* < 0.05, ^##^ *p* < 0.005 and ^###^ *p* < 0.001 compared with the OVA + WPM group; ^+^ *p* < 0.05 and ^+++^ *p* < 0.001 compared with the OVA group.

To confirm the mitigation effect of RL on inflammatory reactions in the lungs exacerbated by PM, we additionally investigated the mRNA expression of MUC5AC and the pro-inflammatory cytokines IL-1β, IL-6, and IL-17A using real-time PCR (Figures 6A–D). The expression of MUC5AC was highest in the OVA + WPM group compared to that in the control and OVA alone groups. However, there was a decreasing tendency when RL was administered orally and intratracheally. Particularly, when administered intratracheally at a high concentration, RL appeared to significantly reduce the expression of MUC5AC. In addition, the expression levels of pro-inflammatory cytokines IL-1β, IL-6, and IL-17A were increased in the OVA group and the OVA + WPM group compared to that of the control group. In particular, the expression in the OVA + WPM group was significantly higher than that in the OVA group. However, when RL was administered orally and intratracheally, the expression level of proinflammatory cytokines exacerbated by WPM decreased in a dose-dependent manner, and when administered at high doses, the level of each cytokine was significantly alleviated.

### *Rosa laevigata* Alleviates Expression of the Mitogen-Activated Protein Kinase Pathway in Mice With Asthma Exacerbated by Water-Soluble Particulate Matter Exposure

**FIGURE 7:**
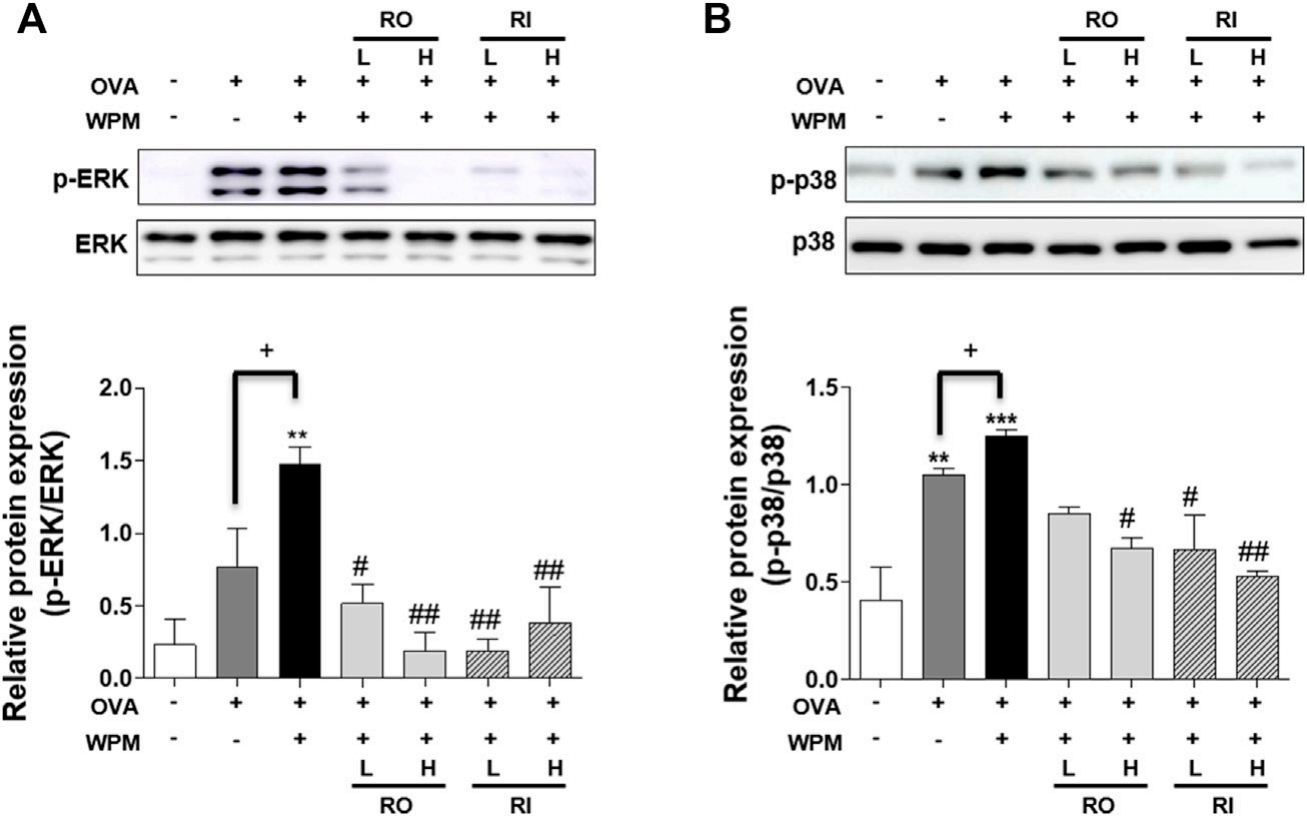
The effects of RL on the MAPK pathway associated protein expression in the lungs of WPM-exacerbated mouse model of asthma. The protein expression level of **(A)** ERK, **(B)** p38 were determined using Western blotting. Western blot results were quantified using Image J software. All data are presented as the mean ± SEM. *n* = 3/group. *p < 0.05 and ***p < 0.001 compared with the control group; ^#^ *p* < 0.05, ^##^ *p* < 0.005 and ^###^ *p* < 0.001 compared with the OVA + WPM group; ^+^ *p* < 0.05 compared with the OVA group.

We investigated the protein expression level of the MAPK signaling pathway related to the Th2 immune response and pro-inflammatory response in a mouse model of asthma exacerbated by WPM using western blotting (Figures 7A,B). Compared with the lung tissue of the control group, phosphorylation of ERK and p38 was significantly increased in the lung tissue of the OVA + WPM group. In contrast, oral and intratracheal administration of RL resulted in a marked decrease in the phosphorylation of ERK and p38 compared to that in mice not treated with RL.

## Discussion

In this study, we investigated whether oral and intratracheal administration of RL could prevent or alleviate allergic reactions and airway and systemic inflammatory reactions, which are asthma symptoms that are worsened by exposure to WPM. This study is the first to investigate the anti-asthmatic effect and treatment mechanism of RL by inhalation and oral administration in a model in which the symptoms of asthma are worsened by exposure to WPM.

To date, studies on the increase in mortality and prevalence from air pollution have continued in developed countries such as the United States and Europe; in particular, PM in the air can cause various respiratory diseases, ischemic heart disease, and cardiovascular disease, depending on air pollutants, ingredients, and concentration (Dominici et al., 2006; Huttunen et al., 2012). The main causes of PM emissions are exhaust gas and fossil fuel combustion. The metal smelting process has recently been reported as the most important emission source of PM, as it increases the inclusion of various heavy metals in high concentrations in fine dust (Zhang H. et al., 2013; Ogundele et al., 2017; Zhang et al., 2018). Recently, studies comparing the different pathological effects of insoluble and soluble PM extracts have been actively conducted to investigate the potential contribution of various components of PM to respiratory diseases (Velali et al., 2016b; Jeong et al., 2017; Zhao et al., 2019a; Zhao et al., 2020a; Wu et al., 2021a). Among these, WPM contains abundant contaminants with significant heavy metal and inorganic ion content. In this study, inductively coupled plasma mass spectrometry (ICP-MS) analysis was performed to investigate the composition of metal components in WPM samples collected from vehicles operating in downtown Seoul. It was found that WPM contains high concentrations of metal elements such as Zn, Fe, Cu, Mn, Al, and Sr. In addition, it contains heavy metals harmful to the human body, such as Pb, As, Cd. Repeated exposure to WPM containing these heavy metals in the nasal cavities of asthmatic mice can cause allergic asthma characterized by eosinophilic and neutrophil infiltration due to pro-inflammatory cell reactions and Th2 cell immune pathway-related inflammatory reactions (Zhao et al., 2019a; Zhao et al., 2020a; Wu et al., 2021a). The histological findings of this study shown through H&E staining similarly showed that the airway epithelium was thicker in mice induced with OVA + WPM than in mice induced with asthma only by OVA, which proves that many inflammatory cells were mobilized to the bronchioles. Furthermore, as a result of confirming the infiltration of inflammatory cells in the airways obtained from BALF by Diff-Quik staining, it was shown that OVA + WPM-induced mice had significantly higher numbers of inflammatory cells, such as macrophages, eosinophils, neutrophils, and lymphocytes, than OVA-induced asthmatic mice. This indicates that the reaction was generated to defend against a more severe inflammatory response due to additional exposure to WPM. However, oral and intratracheal administration of RL significantly reduced the thickness of the lung epithelium caused by inflammatory cell infiltration by attenuating airway inflammation aggravated by WPM. In addition, it significantly reduced the number of inflammatory cells such as macrophages, eosinophils, neutrophils, and lymphocytes. This indicates the potential use of RL as an inhaled and oral treatment for asthma exacerbated by WPM.

Heavy metals are toxic to the immune system, as well as the nervous, cardiovascular, and endocrine systems, and some heavy metals have been reported to activate the secretion of Th2-cell-derived cytokines (Heo et al., 1996; Gavett and Koren, 2001; Gavett et al., 2003; Inoue, 2013; Sankhla et al., 2016). Thus, they exacerbate the onset and symptoms of Th2-related allergic asthma. Cytokines and chemokines related to Th2 cells include IL-4, IL-5, and eotaxin. IL-4 stimulates B cells to increase IgE secretion (Zhang et al., 1991). IL-5 is involved in the differentiation, growth, and activity of immature eosinophils, leading to the infiltration of eosinophils into the lungs (Menzies-Gow et al., 2003). In addition, eotaxin in the blood is a marker of allergic inflammatory reactions and plays a role in inducing eosinophil mobilization from various inflammatory sites (Gutierrez-Ramos et al., 1999). TNF-α, which is involved in the immune system in asthma, is a multifunctional cytokine secreted by the activation of macrophages and is known to promote the secretion of several inflammatory cytokines (Berry et al., 2007). In this study, it was also confirmed that the expression of IL-4, IL-5, and eotaxin was highest in the group with WPM exposure, and TNF-α was rapidly increased by additional exposure to WPM. However, oral and intratracheal administration of RL significantly reduced the levels of Th2-induced cytokines and the pro-inflammatory cytokine TNF-α in the blood, which were increased by WPM. In addition, it has been proven that as the concentration of heavy metals in the blood increases due to exposure to heavy metals, the concentration of IgE in the blood increases, which increases the severity of asthma, bronchial hypersensitivity, and the risk of allergic asthma by constricting smooth muscles (Wang et al., 2017; Wu et al., 2019; Zahedi et al., 2021). Our data showed that the serum IgE concentration was further increased by WPM. In addition, the administration of RL significantly reduced serum IgE levels. Therefore, RL has been proven to alleviate allergic immune responses, consistent with the results of the lowered expression level of Th-2 cytokine in the blood.

Transition metals can increase the level of CD4^+^ cells that are positive for IL-17A, specific to Th17 cells, by monocyte activation (Gałuszka et al., 2020). Studies have also shown that WPM containing transition metals significantly increases the levels of several types of infection-related cytokines, such as IL-1β and IL-6, in the airway epithelium, resulting in airway wall collapse (Zhao et al., 2020a). In the lung tissue, IL-1β and IL-6 stimulate the expression of IL-17A, and IL-17A is involved in allergic airway inflammation and, in particular, airway inflammation caused by neutrophil infiltration (Mizutani et al., 2014). In addition, IL-17A can act with IL-6 to induce MUC5AC, and PM excessively regulates the expression of MUC5AC, causing excessive mucus production in the airways and exacerbating airway obstruction (Chen et al., 2003). Our data confirmed that the mRNA expression of proinflammatory cytokines was significantly increased in the lung tissue of OVA + WPM mice compared to OVA-induced asthma mice, and the expression of MUC5AC also showed a slight tendency to increase. The expression of pro-inflammatory cytokines and MUC5AC in the lungs of RL-treated mice tended to decrease.

MAPK, activated by various stress and inflammatory stimuli, is an important signaling pathway that contributes to the creation and activation of pro-inflammatory mediators in the airway, where resident and invasive cells play an important role (Liu et al., 2008; Wang et al., 2018). The components of the MAPK signaling pathway are representative of three pathways: ERK, p38, and c-Jun NH2-terminal kinase (JNK). To improve airway remodeling due to continuous inflammatory reactions in allergic asthma symptoms, there is a need to develop a treatment that targets and inhibits each specific MAPK pathway, and many studies have been conducted. A common feature of the ERK/p38 signaling pathway is that it is overexpressed in the lungs of asthmatic patients, contributing to the regulation of Th2-related cytokines and chemokines and the activation of eosinophilic differentiation, exacerbating allergic airway inflammation (Nath et al., 2006; Chu et al., 2011). In addition, the ERK pathway may provide a pathway by which IL-17A contributes to mucus production and inflammation in the airways (Inoue et al., 2006). p38 can regulate pro-inflammatory cytokines such as IL-1β and IL-6, and macrophage inflammatory proteins (Bhavsar et al., 2010). As a result of this experiment, the ERK and p38 signaling pathways were more active in asthmatic mice accompanied by WPM than in the OVA alone-induced asthmatic mice, which is consistent with a previous study showing that WPM exposure activates the MAPK pathway in the airway epithelium and worsens airway inflammation. In addition, oral and intratracheal administration of RL significantly blocked phosphorylation of ERK and p38. Therefore, RL is believed to relieve the asthma symptoms exacerbated by WPM induction by interfering with these pathways.

In a previous study, we identified compounds in RL such as quercetin, tormentic acid, and epicatechin, which have strong anti-inflammatory activity, through LC-MS analysis, and they are effective in the treatment of various diseases, including lung disease, by inhibiting the MAPK pathway (Lee et al., 2020). In addition, various flavonoids such as apigenin, ursolic acid, and kaempferol have been isolated and identified from RL, and active ingredients such as saponins also regulate the MAPK pathway and show therapeutic efficacy against various inflammatory diseases (Nguyen et al., 2003; Zhang et al., 2013c; Ma et al., 2014; Malik et al., 2017). Therefore, based on these components, RL is considered to have preventive and therapeutic effects in an asthma model aggravated by WPM induction by regulating the MAPK pathway. However, as RL may show therapeutic efficacy against asthma not only through the MAPK pathway but also through other unknown pathways, further investigation of additional mechanisms is needed.

## Data Availability

The original contributions presented in the study are included in the article/Supplementary Material, further inquiries can be directed to the corresponding author.

## Abbreviations

BALF, Bronchoalveolar lavage fluid; BSA, Bovine serum albumin; COPD, Chronic obstructive pulmonary disease; ECL, Enhanced chemiluminescence; ELISA, Enzyme-linked immunosorbent assay; ERK, Extracellular signal-regulated kinase; H&E, Hematoxylin and eosin; HRP, Horseradish peroxidase; ICP-MS, Inductively coupled plasma mass spectrometry; IgE, Immuno-globulin E; JNK, c-Jun NH2-terminal kinase; MAPK, Mitogen-activated protein kinase; MUC5AC, Mucin 5AC, Oligomeric Mucus/Gel-Forming; NAFLD, Non-alcoholic fatty liver disease; OVA, Ovalbumin; PAH, Polycyclic aromatic hydrocarbon; PCR, Polymerase chain reaction; PM, Particulate matter; RIPA, Radioimmunoprecipitation assay; RL, *Rosa laevigata*; RT, Room temperature; TBST, Tris-buffered saline with Tween 20; Th2, T-helper cell type 2; WPM, Water-soluble particulate matter.

## References

[B1] Berry M. Brightling C. Pavord I. Wardlaw A. (2007). TNF-alpha in Asthma. Curr. Opin. Pharmacol. 7 (3), 279–282. 10.1016/j.coph.2007.03.001 17475560

[B2] Bhavsar P. Khorasani N. Hew M. Johnson M. Chung K. F. (2010). Effect of P38 MAPK Inhibition on Corticosteroid Suppression of Cytokine Release in Severe Asthma. Eur. Respir. J. 35 (4), 750–756. 10.1183/09031936.00071309 19840967

[B3] Bousquet J. Jeffery P. K. Busse W. W. Johnson M. Vignola A. M. (2000). Asthma. From Bronchoconstriction to Airways Inflammation and Remodeling. Am. J. Respir. Crit. Care Med. 161 (5), 1720–1745. 10.1164/ajrccm.161.5.9903102 10806180

[B4] Chen Y. Thai P. Zhao Y. H. Ho Y. S. DeSouza M. M. Wu R. (2003). Stimulation of Airway Mucin Gene Expression by Interleukin (IL)-17 through IL-6 Paracrine/autocrine Loop. J. Biol. Chem. 278 (19), 17036–17043. 10.1074/jbc.M210429200 12624114

[B5] Chu X. Ci X. He J. Wei M. Yang X. Cao Q. (2011). A Novel Anti-inflammatory Role for Ginkgolide B in Asthma via Inhibition of the ERK/MAPK Signaling Pathway. Molecules 16 (9), 7634–7648. 10.3390/molecules16097634 21900866 PMC6264276

[B6] Dominici F. Peng R. D. Bell M. L. Pham L. McDermott A. Zeger S. L. (2006). Fine Particulate Air Pollution and Hospital Admission for Cardiovascular and Respiratory Diseases. Jama 295 (10), 1127–1134. 10.1001/jama.295.10.1127 16522832 PMC3543154

[B7] Dong D. Yin L. Qi Y. Xu L. Peng J. (2015). Protective Effect of the Total Saponins from Rosa Laevigata Michx Fruit against Carbon Tetrachloride-Induced Liver Fibrosis in Rats. Nutrients 7 (6), 4829–4850. 10.3390/nu7064829 26083117 PMC4488818

[B8] Gałuszka A. Stec M. Węglarczyk K. Kluczewska A. Siedlar M. Baran J. (2020). Transition Metal Containing Particulate Matter Promotes Th1 and Th17 Inflammatory Response by Monocyte Activation in Organic and Inorganic Compounds Dependent Manner. Int. J. Environ. Res. Public Health 17 (4), 1227. 10.3390/ijerph17041227 PMC7068527 32074992

[B9] Gavett S. H. Haykal-Coates N. Copeland L. B. Heinrich J. Gilmour M. I. (2003). Metal Composition of Ambient PM2.5 Influences Severity of Allergic Airways Disease in Mice. Environ. Health Perspect. 111 (12), 1471–1477. 10.1289/ehp.6300 12948886 PMC1241649

[B10] Gavett S. H. Koren H. S. (2001). The Role of Particulate Matter in Exacerbation of Atopic Asthma. Int. Arch. Allergy Immunol. 124 (1-3), 109–112. 10.1159/000053685 11306943

[B11] Ge E. Lai K. Xiao X. Luo M. Fang Z. Zeng Y. (2018). Differential Effects of Size-specific Particulate Matter on Emergency Department Visits for Respiratory and Cardiovascular Diseases in Guangzhou, China. Environ. Pollut. 243 (Pt A), 336–345. 10.1016/j.envpol.2018.08.068 30196203

[B12] Gutierrez-Ramos J. C. Lloyd C. Gonzalo J. A. (1999). Eotaxin: from an Eosinophilic Chemokine to a Major Regulator of Allergic Reactions. Immunol. Today 20 (11), 500–504. 10.1016/S0167-5699(99)01522-4 10529777

[B13] Heo Y. Parsons P. J. Lawrence D. A. (1996). Lead Differentially Modifies Cytokine Production *In Vitro* and *In Vivo* . Toxicol. Appl. Pharmacol. 138 (1), 149–157. 10.1006/taap.1996.0108 8658504

[B14] Huttunen K. Siponen T. Salonen I. Yli-Tuomi T. Aurela M. Dufva H. (2012). Low-level Exposure to Ambient Particulate Matter Is Associated with Systemic Inflammation in Ischemic Heart Disease Patients. Environ. Res. 116, 44–51. 10.1016/j.envres.2012.04.004 22541720

[B15] Inoue D. Numasaki M. Watanabe M. Kubo H. Sasaki T. Yasuda H. (2006). IL-17A Promotes the Growth of Airway Epithelial Cells through ERK-dependent Signaling Pathway. Biochem. Biophys. Res. Commun. 347 (4), 852–858. 10.1016/j.bbrc.2006.06.137 16859642

[B16] Inoue K. (2013). Heavy Metal Toxicity. J. Clin. Toxicol. S3, 2161–0495. 10.4172/2161-0495.S3-007

[B17] Jeong S. C. Song M. K. Cho Y. Lee E. Ryu J. C. (2017). Integrative Analysis of mRNA and microRNA Expression of a Human Alveolar Epithelial cell(A549) Exposed to Water and Organic-Soluble Extract from Particulate Matter (PM)2.5. Environ. Toxicol. 32 (1), 302–310. 10.1002/tox.22236 26791009

[B18] Kim K. H. Jahan S. A. Kabir E. (2013). A Review on Human Health Perspective of Air Pollution with Respect to Allergies and Asthma. Environ. Int. 59, 41–52. 10.1016/j.envint.2013.05.007 23770580

[B19] Ko H. M. Choi S. H. Kim Y. An E. J. Lee S. H. Kim K. (2020). Effect of Rosa Laevigata on PM10-Induced Inflammatory Response of Human Lung Epithelial Cells. Evid. Based Complement. Altern. Med. 2020, 2893609. 10.1155/2020/2893609 PMC7492937 32963561

[B20] Lee S.-H. Choi S.-H. Lee I.-S. Kim Y. An E.-J. Jang H.-J. (2020). Anti-inflammatory Effect of Rosa Laevigata Extract on *In Vitro* and *In Vivo* Model of Allergic Asthma via the Suppression of IgE and Related Cytokines. Mol. Cell. Toxicol. 16 (2), 119–127. 10.1007/s13273-019-00063-8

[B21] Li X. Cao W. Shen Y. Li N. Dong X.-P. Wang K.-J. (2012). Antioxidant Compounds from Rosa Laevigata Fruits. Food Chem. 130 (3), 575–580. 10.1016/j.foodchem.2011.07.076

[B22] Liu M. Xu Y. Han X. Liang C. Yin L. Xu L. (2014). Potent Effects of Flavonoid-Rich Extract from Rosa Laevigata Michx Fruit against Hydrogen Peroxide-Induced Damage in PC12 Cells via Attenuation of Oxidative Stress, Inflammation and Apoptosis. Molecules 19 (8), 11816–11832. 10.3390/molecules190811816 25105919 PMC6271498

[B23] Liu W. Liang Q. Balzar S. Wenzel S. Gorska M. Alam R. (2008). Cell-specific Activation Profile of Extracellular Signal-Regulated Kinase 1/2, Jun N-Terminal Kinase, and P38 Mitogen-Activated Protein Kinases in Asthmatic Airways. J. Allergy Clin. Immunol. 121 (4), 893–e2.e892. 10.1016/j.jaci.2008.02.004 18395552

[B24] Liu X. Gao Y. Li D. Liu C. Jin M. Bian J. (2018). The Neuroprotective and Antioxidant Profiles of Selenium-Containing Polysaccharides from the Fruit of Rosa Laevigata. Food Funct. 9 (3), 1800–1808. 10.1039/c7fo01725a 29513319

[B25] Liu Y.-T. Lu B.-N. Xu L.-N. Yin L.-H. Wang X.-N. Peng J.-Y. (2010). The Antioxidant Activity and Hypolipidemic Activity of the Total Flavonoids from the Fruit of Rosa Laevigata Michx. Ns 02 (03), 175–183. 10.4236/ns.2010.23027

[B26] Ma J. Q. Ding J. Zhang L. Liu C. M. (2014). Ursolic Acid Protects Mouse Liver against CCl4-Induced Oxidative Stress and Inflammation by the MAPK/NF-κB Pathway. Environ. Toxicol. Pharmacol. 37 (3), 975–983. 10.1016/j.etap.2014.03.011 24727148

[B27] Malik S. Suchal K. Khan S. I. Bhatia J. Kishore K. Dinda A. K. (2017). Apigenin Ameliorates Streptozotocin-Induced Diabetic Nephropathy in Rats via MAPK-NF-Κb-TNF-α and TGF-Β1-MAPK-Fibronectin Pathways. Am. J. Physiol. Ren. Physiol. 313 (2), F414–F422. 10.1152/ajprenal.00393.2016 28566504

[B28] Menzies-Gow A. Flood-Page P. Sehmi R. Burman J. Hamid Q. Robinson D. S. (2003). Anti-IL-5 (Mepolizumab) Therapy Induces Bone Marrow Eosinophil Maturational Arrest and Decreases Eosinophil Progenitors in the Bronchial Mucosa of Atopic Asthmatics. J. Allergy Clin. Immunol. 111 (4), 714–719. 10.1067/mai.2003.1382 12704348

[B29] Mizutani N. Nabe T. Yoshino S. (2014). IL-17A Promotes the Exacerbation of IL-33-induced Airway Hyperresponsiveness by Enhancing Neutrophilic Inflammation via CXCR2 Signaling in Mice. J. Immunol. 192 (4), 1372–1384. 10.4049/jimmunol.1301538 24446518

[B30] Nath P. Leung S. Y. Williams A. Noble A. Chakravarty S. D. Luedtke G. R. (2006). Importance of P38 Mitogen-Activated Protein Kinase Pathway in Allergic Airway Remodelling and Bronchial Hyperresponsiveness. Eur. J. Pharmacol. 544 (1-3), 160–167. 10.1016/j.ejphar.2006.06.031 16843456

[B31] Nguyen T. T. Tran E. Ong C. K. Lee S. K. Do P. T. Huynh T. T. (2003). Kaempferol-induced Growth Inhibition and Apoptosis in A549 Lung Cancer Cells Is Mediated by Activation of MEK-MAPK. J. Cell. Physiol. 197 (1), 110–121. 10.1002/jcp.10340 12942547

[B32] Ogundele L. T. Owoade O. K. Hopke P. K. Olise F. S. (2017). Heavy Metals in Industrially Emitted Particulate Matter in Ile-Ife, Nigeria. Environ. Res. 156, 320–325. 10.1016/j.envres.2017.03.051 28390299

[B33] Pawankar R. Canonica G. W. Holgate S. T. Lockey R. F. (2012). Allergic Diseases and Asthma: a Major Global Health Concern. Curr. Opin. Allergy Clin. Immunol. 12 (1), 39–41. 10.1097/ACI.0b013e32834ec13b 22157151

[B34] Qu H. Feng Z. Li Z. Li C. Tang M. Zhou Z. (2015). Induction of Substantial Myocardial Regeneration by an Active Fraction of the Chinese Herb Rosa Laevigata Michx. BMC Complement. Altern. Med. 15, 359. 10.1186/s12906-015-0795-0 26467087 PMC4605027

[B35] Rehman A. Amin F. Sadeeqa S. (2018). Prevalence of Asthma and its Management: A Review. J. Pak Med. Assoc. 68 (12), 1823–1827. 30504949

[B36] Sánchez de la Campa A. M. de la Rosa J. D. González-Castanedo Y. Fernández-Camacho R. Alastuey A. Querol X. (2010). High Concentrations of Heavy Metals in PM from Ceramic Factories of Southern Spain. Atmos. Res. 96 (4), 633–644. 10.1016/j.atmosres.2010.02.011

[B37] Sankhla M. S. Kumari M. Nandan M. Kumar R. Agrawal P. (20162016). Heavy Metals Contamination in Water and Their Hazardous Effect on Human Health-A Review. Int. J. Curr. Microbiol. App. Sci. 5 (10), 759–766. 10.20546/ijcmas.2016.510.082

[B38] Schoettler N. Strek M. E. (2020). Recent Advances in Severe Asthma: From Phenotypes to Personalized Medicine. Chest 157 (3), 516–528. 10.1016/j.chest.2019.10.009 31678077 PMC7609962

[B39] Soleimani M. Amini N. Sadeghian B. Wang D. Fang L. (2018). Heavy Metals and Their Source Identification in Particulate Matter (PM2.5) in Isfahan City, Iran. J. Environ. Sci. (China) 72, 166–175. 10.1016/j.jes.2018.01.002 30244743

[B40] Stern J. Pier J. Litonjua A. A. (2020). Asthma Epidemiology and Risk Factors. Semin. Immunopathol. 42 (1), 5–15. 10.1007/s00281-020-00785-1 32020334

[B41] Valavanidis A. Fiotakis K. Vlachogianni T. (2008). Airborne Particulate Matter and Human Health: Toxicological Assessment and Importance of Size and Composition of Particles for Oxidative Damage and Carcinogenic Mechanisms. J. Environ. Sci. Health C Environ. Carcinog. Ecotoxicol. Rev. 26 (4), 339–362. 10.1080/10590500802494538 19034792

[B42] Velali E. Papachristou E. Pantazaki A. Choli-Papadopoulou T. Planou S. Kouras A. (2016a). Redox Activity and *In Vitro* Bioactivity of the Water-Soluble Fraction of Urban Particulate Matter in Relation to Particle Size and Chemical Composition. Environ. Pollut. 208 (Pt B), 774–786. 10.1016/j.envpol.2015.10.058 26586634

[B43] Velali E. Papachristou E. Pantazaki A. Choli-Papadopoulou T. Planou S. Kouras A. (2016b). Redox Activity and *In Vitro* Bioactivity of the Water-Soluble Fraction of Urban Particulate Matter in Relation to Particle Size and Chemical Composition. Environ. Pollut. 208, 774–786. 10.1016/j.envpol.2015.10.058 26586634

[B44] Wang C. Choi Y. H. Xian Z. Zheng M. Piao H. Yan G. (2018). Aloperine Suppresses Allergic Airway Inflammation through NF-Κb, MAPK, and Nrf2/HO-1 Signaling Pathways in Mice. Int. Immunopharmacol. 65, 571–579. 10.1016/j.intimp.2018.11.003 30415164

[B45] Wang I. J. Karmaus W. J. J. Yang C. C. (2017). Lead Exposure, IgE, and the Risk of Asthma in Children. J. Expo. Sci. Environ. Epidemiol. 27 (5), 478–483. 10.1038/jes.2017.5 28401896

[B46] Wong T. W. Lau T. S. Yu T. S. Neller A. Wong S. L. Tam W. (1999). Air Pollution and Hospital Admissions for Respiratory and Cardiovascular Diseases in Hong Kong. Occup. Environ. Med. 56 (10), 679–683. 10.1136/oem.56.10.679 10658547 PMC1757671

[B47] Wu H. Wang D. Shi H. Liu N. Wang C. Tian J. (2021a). PM2.5 and Water-Soluble Components Induce Airway Fibrosis through TGF-β1/Smad3 Signaling Pathway in Asthmatic Rats. Mol. Immunol. 137, 1–10. 10.1016/j.molimm.2021.06.005 34175710

[B48] Wu H. Wang D. Shi H. Liu N. Wang C. Tian J. (2021b). PM2.5 and Water-Soluble Components Induce Airway Fibrosis through TGF-β1/Smad3 Signaling Pathway in Asthmatic Rats. Mol. Immunol. 137, 1–10. 10.1016/j.molimm.2021.06.005 34175710

[B49] Wu K. G. Chang C. Y. Yen C. Y. Lai C. C. (2019). Associations between Environmental Heavy Metal Exposure and Childhood Asthma: A Population-Based Study. J. Microbiol. Immunol. Infect. 52 (2), 352–362. 10.1016/j.jmii.2018.08.001 30177433

[B50] Zahedi A. Hassanvand M. S. Jaafarzadeh N. Ghadiri A. Shamsipour M. Dehcheshmeh M. G. (2021). Effect of Ambient Air PM2.5-bound Heavy Metals on Blood Metal(loid)s and Children's Asthma and Allergy Pro-inflammatory (IgE, IL-4 and IL-13) Biomarkers. J. Trace Elem. Med. Biol. 68, 126826. 10.1016/j.jtemb.2021.126826 34371327

[B51] Zhang H. Luo Y. Makino T. Wu L. Nanzyo M. (2013a). The Heavy Metal Partition in Size-Fractions of the Fine Particles in Agricultural Soils Contaminated by Waste Water and Smelter Dust. J. Hazard Mater 248-249, 303–312. 10.1016/j.jhazmat.2013.01.019 23416473

[B52] Zhang K. Chai F. Zheng Z. Yang Q. Zhong X. Fomba K. W. (2018). Size Distribution and Source of Heavy Metals in Particulate Matter on the Lead and Zinc Smelting Affected Area. J. Environ. Sci. (China) 71, 188–196. 10.1016/j.jes.2018.04.018 30195677

[B53] Zhang K. Clark E. A. Saxon A. (1991). CD40 Stimulation Provides an IFN-gamma-independent and IL-4-dependent Differentiation Signal Directly to Human B Cells for IgE Production. J. Immunol. 146 (6), 1836–1842. 1706382

[B54] Zhang S. Qi Y. Xu Y. Han X. Peng J. Liu K. (2013c). Protective Effect of Flavonoid-Rich Extract from Rosa Laevigata Michx on Cerebral Ischemia-Reperfusion Injury through Suppression of Apoptosis and Inflammation. Neurochem. Int. 63 (5), 522–532. 10.1016/j.neuint.2013.08.008 24012531

[B55] Zhang S. Qi Y. Xu Y. Han X. Peng J. Liu K. (2013d). Protective Effect of Flavonoid-Rich Extract from Rosa Laevigata Michx on Cerebral Ischemia-Reperfusion Injury through Suppression of Apoptosis and Inflammation. Neurochem. Int. 63 (5), 522–532. 10.1016/j.neuint.2013.08.008 24012531

[B56] Zhang S. Zheng L. Dong D. Xu L. Yin L. Qi Y. (2013e). Effects of Flavonoids from Rosa Laevigata Michx Fruit against High-Fat Diet-Induced Non-alcoholic Fatty Liver Disease in Rats. Food Chem. 141 (3), 2108–2116. 10.1016/j.foodchem.2013.05.019 23870935

[B57] Zhang S. Lu B. Han X. Xu L. Qi Y. Yin L. (2013b). Protection of the Flavonoid Fraction from Rosa Laevigata Michx Fruit against Carbon Tetrachloride-Induced Acute Liver Injury in Mice. Food Chem. Toxicol. 55, 60–69. 10.1016/j.fct.2012.12.041 23279844

[B58] Zhao C. Niu M. Song S. Li J. Su Z. Wang Y. (2019a). Serum Metabolomics Analysis of Mice that Received Repeated Airway Exposure to a Water-Soluble PM2.5 Extract. Ecotoxicol. Environ. Saf. 168, 102–109. 10.1016/j.ecoenv.2018.10.068 30384157

[B59] Zhao C. Niu M. Song S. Li J. Su Z. Wang Y. (2019b). Serum Metabolomics Analysis of Mice that Received Repeated Airway Exposure to a Water-Soluble PM2.5 Extract. Ecotoxicol. Environ. Saf. 168, 102–109. 10.1016/j.ecoenv.2018.10.068 30384157

[B60] Zhao C. Wang Y. Su Z. Pu W. Niu M. Song S. (2020a). Respiratory Exposure to PM2.5 Soluble Extract Disrupts Mucosal Barrier Function and Promotes the Development of Experimental Asthma. Sci. Total Environ. 730, 139145. 10.1016/j.scitotenv.2020.139145 32402975

[B61] Zhao C. Wang Y. Su Z. Pu W. Niu M. Song S. (2020b). Respiratory Exposure to PM2.5 Soluble Extract Disrupts Mucosal Barrier Function and Promotes the Development of Experimental Asthma. Sci. Total Environ. 730, 139145. 10.1016/j.scitotenv.2020.139145 32402975

[B62] Zhao L. Xu L. Tao X. Han X. Yin L. Qi Y. (2016). Protective Effect of the Total Flavonoids from Rosa Laevigata Michx Fruit on Renal Ischemia-Reperfusion Injury through Suppression of Oxidative Stress and Inflammation. Molecules 21 (7), 952. 10.3390/molecules21070952 PMC6272996 27455216

