# Supplementary material for: Rosa laevigata Attenuates Allergic Asthma Exacerbated by Water-Soluble PM by Downregulating the MAPK Pathway
Source: Front Pharmacol. 2022 Jun 28;13:925502. doi: 10.3389/fphar.2022.925502 (PMC9274115; doi:10.3389/fphar.2022.925502)
Supplement: Supplementary file 1 [file Table1.DOCX]

# Supplementary Data

Table. S1 Measurement of component detected by ICP-MS in WPM

| **Metal** | **WPM**  **(**mg/kg**)** | **Control filter (**mg/kg**)** |
| --- | --- | --- |
| Zn | 3155.11 | N.D. |
| Fe | 1226.41 | 2.78 |
| Cu | 1153.34 | 0.08 |
| Mn | 866 | N.D. |
| Al | 724.55 | 0.15 |
| Sr | 262.18 | 0.11 |
| Ti | 83.46 | N.D. |
| Mo | 47.49 | N.D. |
| Ni | 34.52 | N.D. |
| As | 19.81 | N.D. |
| Cr | 15.34 | N.D. |
| Pb | 12.95 | N.D. |
| Co | 8.99 | N.D. |
| Se | 8.25 | N.D. |
| Cd | 4.15 | N.D. |
| Be | 0.05 | N.D. |
